# Enhancing Capacitance Performance of Ti_3_C_2_T_*x*_ MXene as Electrode Materials of Supercapacitor: From Controlled Preparation to Composite Structure Construction

**DOI:** 10.1007/s40820-020-0415-5

**Published:** 2020-03-20

**Authors:** Xiaobei Zang, Jiali Wang, Yijiang Qin, Teng Wang, Chengpeng He, Qingguo Shao, Hongwei Zhu, Ning Cao

**Affiliations:** 1 School of Materials Science and Engineering, China University of Petroleum (East China), Qingdao, 266580 People’s Republic of China; 2 School of Materials Science and Engineering, Tsinghua University, Beijing, 100084 People’s Republic of China

**Keywords:** Ti_3_C_2_T_*x*_, MXene, Capacitance performance, Storage mechanism, Electrode materials, Supercapacitor

## Abstract

Ti_3_C_2_T_*x*_, a novel two-dimensional layer material, is widely used as electrode materials of supercapacitor due to its good metal conductivity, redox reaction active surface, and so on. However, there are many challenges to be addressed which impede Ti_3_C_2_T_*x*_ obtaining the ideal specific capacitance, such as restacking, re-crushing, and oxidation of titanium. Recently, many advances have been proposed to enhance capacitance performance of Ti_3_C_2_T_*x*_. In this review, recent strategies for improving specific capacitance are summarized and compared, for example, film formation, surface modification, and composite method. Furthermore, in order to comprehend the mechanism of those efforts, this review analyzes the energy storage performance in different electrolytes and influencing factors. This review is expected to predict redouble research direction of Ti_3_C_2_T_*x*_ materials in supercapacitors.

## Highlights

- The traditional and novel etching methods are summarized and compared, especially fluorine-free method. The methods for accelerating exfoliation of Ti_3_C_2_T_*x*_ are classified.
- The energy storage mechanisms of Ti_3_C_2_T_*x*_ in different electrolytes are compared. Based on energy storage mechanisms, the influencing factors of morphology and surface functional groups are discussed.
- In response to the problems of the Ti_3_C_2_T_*x*_, strategies for improving capacitance from structure modulation to composite structure construction are summarized and compared.

## Graphical abstract

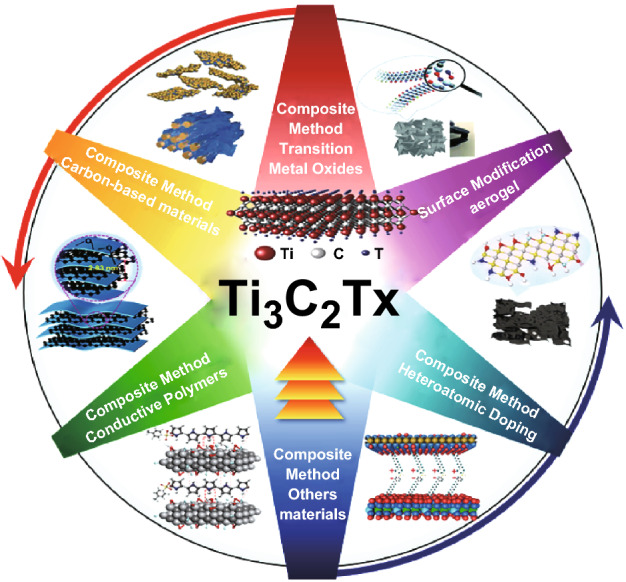

## Introduction

With the deterioration of environment and the depletion of traditional fossil energy, renewable and sustainable energy has been attracted much attention [1, 2]. However, the supply of these kinds of energy is intermittent, due to their dependence on weather. As supplementary, energy storage devices have drawn tremendous interest recently [3, 4]. Supercapacitors, one of new energy storage devices between batteries and traditional parallel-plate capacitors, have the characteristic of high power density, rapid charge/discharge rate, and excellent cycle stability [5, 6]. Electrode materials are one of the critical factors affecting the capacitance performance of supercapacitors. The common electrode materials include carbon-based materials [7–9], transition metal oxides (TMOs) [10], conductive polymers (CPs), and so on [11–14]. Nevertheless, the prior researches have proved that the low density of carbon-based materials, the poor conductivity of TMOs, and the instable structure of CPs hinder them from achieving ideal capacitance performance [15, 16]. Therefore, there is still a significant challenge to improve the performance of electrode materials of supercapacitors for practical use.

In 2011, an interesting two-dimensional material named MXene was synthesized by Gogosti [17]. MXene is a novel two-dimensional (2D) layered material composed of transition metal carbides and/or nitrides, and it is formed through selectively etching the A layers from MAX precursor, where M represents transition metals, such as Ti, Zr, Nb, Ta, and Mo and X represents carbon and/or nitrogen [18–21]. Many MXene materials have been studied recently, such as V_2_C_2_T_*x*_, Ti_3_C_2_T_*x*_, and Ti_2_CT_*x*_. [22, 23]. In the formula, T_*x*_ is the surface functional group introduced by etching condition, such as –O, –F, and –OH [24, 25]. Due to its low formation energy, Ti_3_C_2_T_*x*_ is the first and one of the most widely studied MXene materials [26]. Ti_3_C_2_T_*x*_ overcomes the shortcomings in general electrode materials, and it has become a hot spot in supercapacitors. Even so, there are still many problems to be solved, as follows: (1) Ti_3_C_2_T_*x*_ has ultrahigh volume specific capacitance ascribed to its high density (~ 3.8 g cm^−3^ in Ti_3_C_2_T_*x*_ film) [27]. However, the self-restacking caused by the van der Waals force restricts active surface utilization. Besides the irreversible stacking between Ti_3_C_2_T_*x*_ layers, the aggregation resulting from the effect of hydrogen bonding also restrains the redox reaction of Ti_3_C_2_T_*x*_ edges [28, 29]; (2) structure stability is a critical factor influencing the cycle performance. During the charge and discharge, it is unavoidable to incur the volume change, leading to large structural stress. If the stress exceeds the strength of Ti_3_C_2_T_*x*_, it is clear that structural deformation and damage will happen, which are manifested by the lamellar re-crushing or shedding of active materials from electrode [30]; (3) the adsorption of oxygen or water molecules may partially oxidize Ti_3_C_2_T_*x*_ to non-conductive titanium dioxide (TiO_2_), which will reduce redox reaction active sites and increase the charge transfer impendence [30, 31]. This defect is particularly significant in the hydrothermal method [32].

**Fig. 1:**
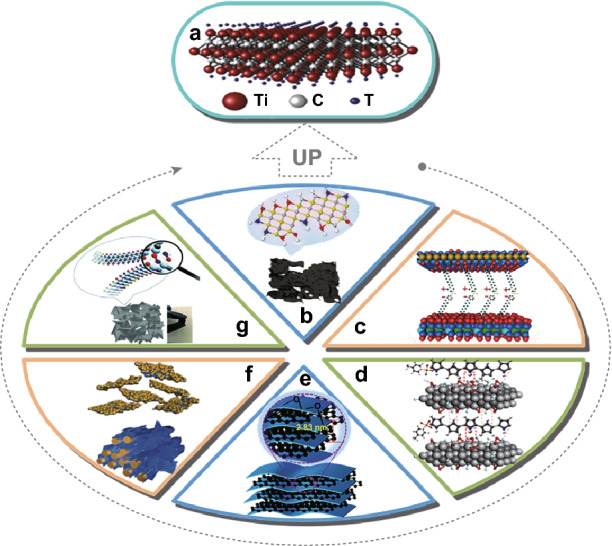
Enhancing the capacitance of Ti_3_C_2_T_*x*_ as electrode materials of supercapacitors. **a** Structure of Ti_3_C_2_T_*x*_ [21]. **b** N-doped Ti_3_C_2_T_*x*_ [36]. **c** Ti_3_C_2_T_*x*_/layered metallic double hydroxides [37]. **d** Ti_3_C_2_T_*x*_/conductive polymers [41]. **e** Carbon-intercalated Ti_3_C_2_T_x_ composite paper [38]. **f** WO_3_/Ti_3_C_2_T_*x*_ composite paper [39]. **g** 3D Ti_3_C_2_T_*x*_ aerogels [40]. Reproduced with permission from Refs. [21, 36–40]

In order to control those situations, many works have focused on the modification of Ti_3_C_2_T_*x*_ [33–35]. Figure 1 shows the common methods of enhancing the capacitance of Ti_3_C_2_T_*x*_ as electrode materials of supercapacitors [21, 36–41]. With the efforts of predecessors, many interesting ideas about enhancing capacitance have been put forward, such as modification of preparation process, surface modification, and composite methods. The self-stacking obstacle can endure by increasing the layer space, pillaring effect, and other measurements. In addition, one of the strategies for maintaining structural stability is to increase the strength of the materials. Wang et al. [16] prepare a vacuum-filtered Ti_3_C_2_T_*x*_/PDA film after dopamine self-polymerized on the Ti_3_C_2_T_*x*_ nanosheets into the layered polydopamine (PDA). The interlayer of PDA increases the layer space of Ti_3_C_2_T_*x*_ solving the self-stacking during cycling. Dopamine forms hydrogen bonding with surface functional groups, while Ti forms strong bonding with oxygen atoms in polydopamine, which help strengthen the materials and maintain the stability of the structure [16]. Naguib et al. [42] mixed the Ti_3_C_2_T_*x*_ with polyacrylamide (PAM) and dried it into composite film, where the Ti_3_C_2_T_*x*_ nanoflakes are dispersed in a network formed by PAM. Placing Ti_3_C_2_T_*x*_ in the 3D network also helps maintain structural stability. The oxidation of Ti_3_C_2_T_*x*_ is avoided through controlling the temperature and using reducing atmosphere in the laboratory [43, 44].

In this review, we devote to the brief summarization of the approaches for improving the electrochemical property of Ti_3_C_2_T_*x*_ as the electrode materials of supercapacitors, including the preparation process, surface terminations, precursor, electrolyte, and other governing factors. We hope that it will provide the guidance for further research of Ti_3_C_2_T_*x*_ in supercapacitors.

## Properties of Ti_3_C_2_T_*x*_

**Fig. 2:**
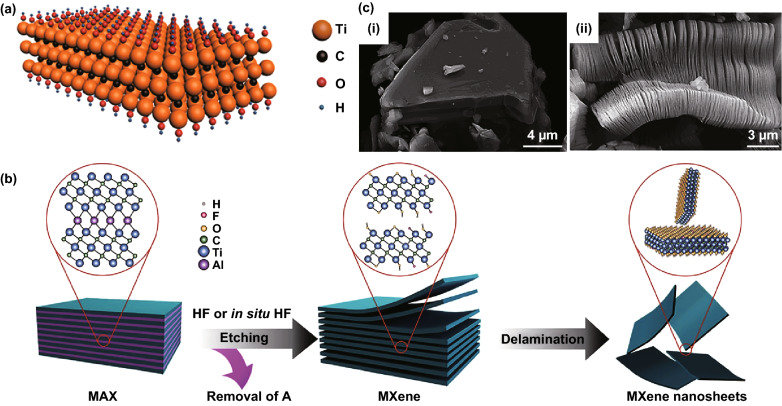
Structure of Ti_3_C_2_T_*x*_. **a** Atomic composition model of Ti_3_C_2_T_*x*_ [25]. **b** Schematic diagram of the process for etching and delamination of Mxene [51]. **c** Scanning electron microscopy (SEM) images of Ti_3_AlC_2_ particle and Ti_3_C_2_T_*x*_ [23]. Reproduced with permission from Refs. [23, 25, 51]

As the representative candidate of electrode materials, Ti_3_C_2_T_*x*_ owns the 2D structure like famous materials—graphene. The atomic composition model is shown in Fig. 2a [25]. In contrast to carbon materials, Ti_3_C_2_T_*x*_ contains metal element contributing to high density and surface functional group simultaneously. On account of its special composition, Ti_3_C_2_T_*x*_ is provided with unique characteristics. Owing to the high density of Ti_3_C_2_T_*x*_ compared with carbon-based materials, its volumetric capacitance is higher. Ti_3_C_2_T_*x*_ has metal conductivity combined with hydrophilicity which guarantees the fast electron transfer and wetting of electrolyte. The adjustable surface functional groups T_*x*_ lead to a change in outward structure to meet different needs. T_*x*_, particularly –O, on the surface of Ti_3_C_2_T_*x*_ provide many active sites for redox reaction [45]. Furthermore, the surface is negatively charge due to the negative surface functional groups, and it behaves hydrophilicity through hydrogen bonding between water and T_*x*_, contributing to the good dispersibility in aqueous solution [29, 46]. Normally, the oxidation of Ti_3_C_2_T_*x*_ is controlled by external temperature. Therefore, it is of great significance to study its thermal stability. When Ti_3_C_2_T_*x*_ is heated, the changes are completed in four stages. As the temperature increases, interlayer water (100 °C), –OH (200–400 °C), and –F (500–800 °C) are gradually removed, eventually resulting in the carbonization of Ti_3_C_2_T_*x*_ [47].

Stemming from its unique structure and properties, Ti_3_C_2_T_*x*_ has been considered the candidate to amend imperfections of common electrode materials of energy storage, except to water purification [48, 49], photo-electrocatalyst, and electromagnetic shielding [50, 51]. Ti_3_C_2_T_*x*_ can be used as reinforcement to improve electrochemical properties of other materials, such as CPs and TMOs [42, 52]. Zhu et al. utilized electrophoretic deposition intercalated polypyrrole (PPy) in Ti_3_C_2_T_*x*_, forming interconnected structure [41]. The stabilized layered structure of Ti_3_C_2_T_*x*_ improves the structural stability of PPy composites impeding the volume contraction/expansion during cycling. Moreover, Ti_3_C_2_T_*x*_ as a substrate for growth of MnO_2_ particles ensures its uniform distribution and the good metal conductivity of Ti_3_C_2_T_*x*_ to provide the conditions for rapid electron transport of the hybrid films [53].

## Preparation of Ti_3_C_2_T_*x*_

There are two strategies to synthesize 2D Ti_3_C_2_T_*x*_: top-down and bottom-up methods. In this review, we discuss some common top-down methods, such as HF-etching, LiF–HCl etching, and fluoride-free etching. Generally, the synthesis of Ti_3_C_2_T_*x*_ includes four main steps, as shown in Fig. 2b [51]: preparation of Ti_3_AlC_2_ (Fig. 2c(i)) [23], etching Al layer, intercalation, and exfoliation. The common etching measurements are implemented by HF-etching and LiF–HCl etching, as showing in Eq. 1 [17, 54]. Then, the surface of 2D Ti_3_C_2_ is covered by –O, –OH, and –F forming Ti_3_C_2_T_*x*_.

1 $${\text{Ti}}_{3} {\text{AlC}}_{2} + 3{\text{H}}^{ + } + 3{\text{F}}^{ - } = {\text{AlF}}_{3} + 3/2{\text{H}}_{2} + {\text{Ti}}_{3} {\text{C}}_{2}$$

The main difference between the two methods is that the latter one has lithium ion intercalation during etching, which both increases the distance between Ti_3_C_2_T_*x*_ layers, the area of Ti_3_C_2_T_*x*_ flakes, and the number of hydroxyl functional groups. HF-etching requires the cation intercalation to assist in delaminating Ti_3_C_2_T_*x*_ into single or few layer sheets, and the morphology of HF–Ti_3_C_2_T_*x*_ is shown in Fig. 2c(ii) [23]. And HF–Ti_3_C_2_T_*x*_ has small hole defects that makes active titanium ion expose to the air and aggravates the oxidation process, forming TiO_2_. LiF–HCl etching method is also called the clay method, because of clay-like behavior of LiF–HCl–Ti_3_C_2_T_*x*_. LiF–HCl–Ti_3_C_2_T_*x*_ is hydrophilic owing to abundant –OH. In the etching process, the volume expanses almost twice, as a consequence of the hydration of intercalated lithium [55]. The extent of volume expansion depends on the hydration enthalpy or hydration radius of the cation. Besides, the etching trace is in positive correlation with the content of LiF and HCl, where lithium ion intercalation depends on the former, while the degree of reaction depends on the latter. Compared with the HF-etching method, the LiF–HCl etching method is milder and the etching product has higher surface quality and better mechanical stability, since the size of the nanosheet is larger without ultrasonic treatment in the exfoliating process.

In addition, the morphology of Ti_3_C_2_T_*x*_ is affected by the etching temperature, etching time, ball milling time of precursors in both etching methods. For example, surface functional groups introduced by etching inhibit further etching of Al layer; the increasing etching temperature accelerates the etching rate and degree [56]; the conductivity is better when the ball milling time is longer, which causes the greater destroy of interlayer van der Waals force and the more exposure of the carbon ions [57]. However, when the etching time is too long, Ti_3_C_2_T_*x*_ sheets will be corroded into holes or even damaged for the corrosion of titanium atoms [58].

There are the other etching methods to obtain Ti_3_C_2_T_*x*_ sheets. Many studies focus on avoiding the use of fluorine-containing reagents. Yang et al. [59] prepared Ti_3_C_2_T_*x*_ by an electrochemical etching method, using ammonium chloride (NH_4_Cl) as the electrolyte and Ti_3_AlC_2_ as the anode in a particular voltage range. Since the intensity of Al–Cl bond is stronger than that of the Al–Ti, the Al can be etched by Cl^−^ to form Al–Cl. Furthermore, the open structure is formed by ammonium ions intercalated uniformly. The increase in layer space avails the permeation of Cl^−^, accelerating the etching process. Specially, the structure of Ti_3_C_2_T_*x*_ in this study is dense instead of the common accordion shape, because less gas is released during etching. Li et al. successfully build the 2D Ti_3_C_2_T_*x*_ nanosheets by KOH-etching [60]. In this method, KOH mixed with small amounts water reacts with Al layers releasing H_2_. Meanwhile, the extraction of Al layer leads to the adhering of –OH during the etching and exfoliation process. The merits of those fluoride-free etching process compared with the traditional methods (HF-etching and LiF–HCl etching) include operational performance and capacitance performance of Ti_3_C_2_T_*x*_. As we all know, HF is one of the most dangerous reagents in the laboratory. As a result, one of the advantages of this fluoride-free process is to avoid the safety hazards of using HF-etching or in-situ HF-etching; the other is that the prepared Ti_3_C_2_T_*x*_ does not contain –F, which harms the specific capacitance. The effects of –F are discussed later in detail. Interestingly, the main purpose of etching is to remove the Al layer while maintaining the original layered structure. But some researches indicate the appropriate residue of Al will improve the conductivity of Ti_3_C_2_T_*x*_. Guo et al. [30] found that a part of Al layers is retained using hydrothermal method with the intercalation of potassium ions. It is beneficial for Ti_3_C_2_T_*x*_ to maintain a stable layered structure. The retained Al elements form electron transfer channels to improve conductivity. Meanwhile, the space left by removing Al elements is favorable for the penetration of electrolyte ions in supercapacitors.

**Fig. 3:**
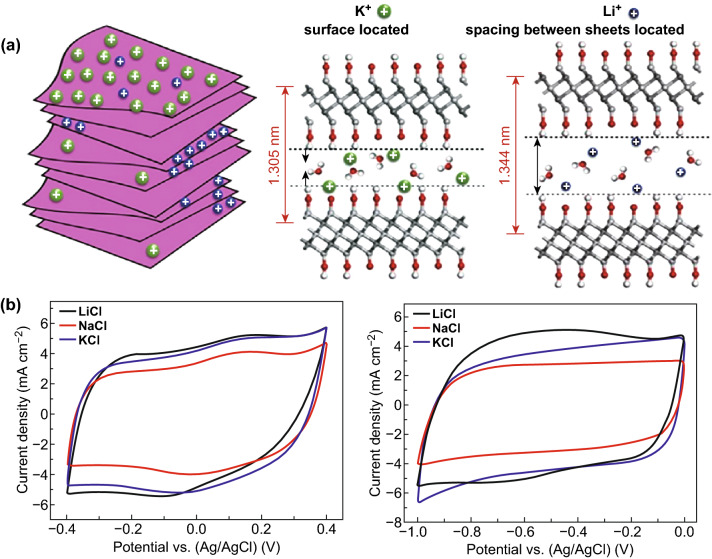
Electrochemical performance of Ti_3_C_2_T_*x*_ at a scan rate of 5 mV s^−1^. **a** Schematic illustration of ionic intercalation mechanism on the surface of the Ti_3_C_2_T_*x*_. **b** CV curves of Ti_3_C_2_T_*x*_ in aqueous LiCl, NaCl, and KCl electrolyte at different potential windows. Reproduced with permission from Ref. [64]

The exfoliation is a necessary process for obtaining single layer or few layers of Ti_3_C_2_T_*x*_. At present, there are many methods for accelerating the exfoliation of materials, including: (1) mechanical methods: Feng et al. [56] showed the ultrasonic treatment-assisted exfoliating process could inhibit the formation of a stable force between layers to obtain Ti_3_C_2_T_*x*_ sheets with rich functional groups on the surface, but also cause the problem of a small lateral dimension. (2) The introduction of repulsion groups: Qian et al. [61] revealed that the repulsion between the methyl group of dimethyl sulfoxide (DMSO) organic intercalant and the hydroxyl group on the surface of Ti_3_C_2_T_*x*_ contributes to increase the interlayer spacing. Meanwhile, –F is replaced partly by a hydroxyl group during the exfoliating, leaving the high boiling organic reagent [62]. As a result, it is difficult to remove impurities. (3) The hydrothermal method: It is well known that the increase in the temperature of solvent can heighten the energy of the intercalated ions, which accelerates the diffusion of the intercalation reagent to break the energy barrier of the Ti–Ti bond, but the oxidation phenomenon of Ti_3_C_2_T_*x*_ tends to increase during the hydrothermal reaction [63]. It is necessary to add antioxidant for inhibiting the process. (4) The ion-assisted intercalation: The TMAOH polar molecules are candidates for intercalation materials, because it is easy to enter the interlayer van de Waals force and react with the remaining Al. The formed aluminum hydroxide can further increase the interlayer spacing. Ion-assisted intercalation, an important exfoliating method, mainly depends on the diameter of the ions and the relative size of the layer spacing. That is the reason why the intercalation efficiency of Li ions is significantly higher than that of other metal cations, as shown in Fig. 3a [64]. It should be noted that the material avoids attaching at the edge of Ti_3_C_2_T_*x*_ as a consequence of large diameters.

## Capacitance Properties

According to the energy storage mechanism, the supercapacitor can be divided into two categories: (1) electric double-layer capacitor, in which ions are adsorbed by an electrical layer between the electrolyte and the electrode [65, 66]; (2) pseudocapacitance, which utilizes a rapid Faradic reaction occurring on the surface of the electrode to store energy [67].

With regard to Ti_3_C_2_T_*x*_, it exhibits different capacitance characteristics, which depends on the size of electrolyte cations, the functional groups, the status of interlayer water, the morphological structure, and so on. In particular, the Ti_3_C_2_T_*x*_ nanosheet is negatively charged with negative functional groups like –F, –O, and –OH. When electrolyte cations move in Ti_3_C_2_T_*x*_ layers, it produces the electrostatic attraction between opposite charges. At this time, if the electrolyte cation is small enough to pass through Ti_3_C_2_T_*x*_ layers, which causes the electrode to be deformed, additional pseudocapacitance is generated. Conversely, the larger ions cannot penetrate into layers and can only form electrostatic repulsion at the edge of the intercalation layer to maintain high cycle stability, and the electric double-layer capacitance is formed by electrochemical adsorption.

### Electric Double-Layer Capacitance

Electric double-layer capacitor is similar to traditional parallel capacitor in that it forms an electrical layer between the electrode and electrolyte to store energy, where the polar solvent between the electrode and ions serves as the electrolyte [68, 69]. This formation characteristic determines that the specific surface area of the electrode material affects the amount of ions absorbed. If the surface area increases or the distance of ions diffusion reduces, the specific capacitance of Ti_3_C_2_T_*x*_ may be enhanced. This may provide a new opinion for enhancing the capacitance of Ti_3_C_2_T_*x*_ in further research. In neutral aqueous electrolyte or organic electrolyte, the energy storage mechanism of Ti_3_C_2_T_*x*_ may behave as electric double-layer capacitance [27, 64]. Moreover, whether electric double-layer capacitance can be formed in the Ti_3_C_2_T_*x*_ depends on the diameter of electrolyte ions and the relative size of interlayer spacing. If the diameter of electrolyte ions is smaller than the layer space, the energy is stored by intercalation instead of electroadsorption. Qian et al. study the change of capacitance associated with cation [60]. As shown in Fig. 3b, the CV curves of electric double-layer capacitance are typical rectangular profiles without redox peak [64] and there is no obvious voltage drop in the beginning of the charge and discharge. At the same time, the specific capacitance contributed to electric double-layer capacitance is mainly controlled by ionic concentration. Xia et al. using a simulated seawater solution (0.6 M NaCl) measured Ti_3_C_2_T_*x*_ which has specific capacitance of only 67.7 F g^−1^ (1 A g^−1^) [70]. Although this type of electrolyte is low in cost and high in safety, we can infer from the capacitance performance that a small number of ions leads to low ion conductivity and a few adsorbed ions. Compared with pseudocapacitance, this energy storage mechanism is not extensive, so there are few related investigations.

### Pseudocapacitance

#### The Influence of Electrolyte

##### Aqueous Electrolyte

**Fig. 4:**
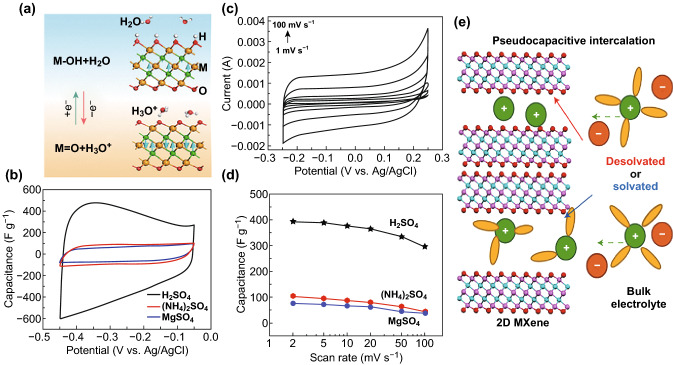
Pseudocapacitance of Ti_3_C_2_T_*x*_ in different electrolytes. **a** The change of surface group of Ti_3_C_2_T_*x*_ in H_2_SO_4_ [58]. **b** CV curves of Ti_3_C_2_T_*x*_ at a scan rate of 20 mV s^−1^ [71]. **c** Gravimetric capacitances of Ti_3_C_2_T_*x*_ at different scan rates [75]. **d** CV curves of Ti_3_C_2_T_*x*_ at different scan rates in KOH electrolyte [75]. **e** Schematic of supercapacitor using Ti_3_C_2_T_*x*_ (pink, Ti; cyan, C; red, O) as negative electrode with solvated or desolvated states. Legend for the electrolyte: green, cation; orange, anion; yellow, solvent molecule [81]. Reproduced with permission from Refs. [58, 71, 75, 81]. (Color figure online)

One of the most common aqueous electrolytes is sulfuric acid electrolyte. Since the cation in the sulfuric acid electrolyte is a small-sized hydrogen ion, it is permeable to the nanosheet layer and causes high ionic conductivity. When Ti_3_C_2_T_*x*_ is in the sulfuric acid electrolyte, the storage mechanism of pseudocapacitance is dominant [71, 72]. The intercalation of hydrogen ions protonates the oxygen functional group on the surface, forming a hydroxyl group (Fig. 4a, b) [58, 71], and the oxidation state of Ti is changed. The reversible change of the Ti oxidation valence state from +3 to +4 follows bonding and bond-breaking of the oxygen functional group, respectively [73]. Briefly, the above electrochemical reaction can be described by Eqs. 2 and 3:

2 $${\text{Ti}}_{3} {\text{C}}_{2} {\text{O}}_{\text{x}} +\updelta{\text{e}}^{ - } +\updelta{\text{H}}^{ + } = {\text{Ti}}_{3} {\text{C}}_{2} {\text{O}}_{{{\text{x}} -\updelta}} \left( {\text{OH}} \right)_{\updelta}$$

3 $${\text{Ti}}_{3} {\text{C}}_{2} {\text{O}}_{{\rm x}} \left( {\text{OH}} \right)_{{\rm y}} {\text{F}}_{{\rm z}} +\updelta{\text{e}}^{ - } +\updelta{\text{K}}^{ + } = {\text{K}}_{\updelta} {\text{Ti}}_{3} {\text{C}}_{2} {\text{O}}_{{\rm x}} \left( {\text{OH}} \right)_{{\rm y}} {\text{F}}_{{\rm z}}$$

It can be seen from Eq. 2 that the increasing concentration of sulfuric acid further improves the ion conductivity of the electrolyte to enhance the specific capacitance of supercapacitors.

Similar to acid electrolyte, alkaline electrolyte (KOH) could provide ion intercalation, without the reaction of surface functional groups, as shown in Eq. 3, and Fig. 4c shows its CV curves [74, 75]. Li^+^, as the metal cation with minimum radius, is a frequently used neutral electrolyte ion, such as Li_2_SO_4_ aqueous solution. In the cycling process, the redox reaction occurs with the reversible intercalation/deintercalation of Li^+^. By comparison, the usage of neutral water electrolytes could improve the safety of supercapacitors. However, the oxidation of Ti_3_C_2_T_*x*_ occurs when the potential of Ti_3_C_2_T_*x*_ is high in aqueous electrolyte (for example, anodic oxidation when potential is over 0.6 V) [76]. As a result, it limits the expansion of voltage window of aqueous electrolyte and the energy density [77].

##### Ionic Liquid

The application of ionic liquid electrolyte helps Ti_3_C_2_T_*x*_ to solve this problem. Ionic liquid, a kind of molten salt, consists of organic cations and organic or inorganic anions, which keeps its liquid state at or near room temperature [78]. Jackel et al. studied the volume change of Ti_3_C_2_T_*x*_ in ionic liquids by means of electrochemical tracing method [79]. The results show that the volume expansion of Ti_3_C_2_T_*x*_ in ionic liquids is irreversible due to the spontaneous ion intercalation when the electrodes contact the electrolytes of ionic liquids. Volume expands at negative potential during electrochemical cycling because of the negative charge on the surface of Ti_3_C_2_T_*x*_, and the intercalation of cations is enhanced to maintain electrostatic equilibrium. Ion delamination and volume shrinkage of electrostatic adsorption occur at positive potential, and the permeation of solvents also has a certain effect on volume change.

In addition, the humidity of the environment causes water molecules to replace the ions on the surface of Ti_3_C_2_T_*x*_ to increase the fluidity of the ionic liquid, so the use of this electrolyte requires humidity control [80]. The non-aqueous electrolyte solution often has a problem of poor conductivity due to large ion size, which affects the intercalation effect.

##### Organic Electrolyte

Gogotsi et al. [81] used the lithium ion intercalation behavior of lithium hexafluorophosphate in various solvents to study the effect of electrolyte solvent on the capacitance performance. Experiments showed that the desolvation of Li intercalation is beneficial to extend the voltage window to 2.4 V and improve the capacitance performance of Ti_3_C_2_T_*x*_. If organic solvent intercalates with Li ions, the intercalation efficiency of Li ions is reduced (Fig. 4d) [81]. At the same time, the ion conductivity of the solvent also affects the permeation of ions. Oxygen in DMSO solvent, which cannot be desolvated interacts with Li ion, keeping the hydrophobic methyl away from the surface of Ti_3_C_2_T_*x*_. Li ion intercalation weakens the electrostatic interaction between layers.

#### The Influence of Morphology

In light of the changes in the charge and discharge process of Ti_3_C_2_T_*x*_ in various electrolytes, the structure and morphology of Ti_3_C_2_T_*x*_ also affect the capacitance performance. Sugahara et al. [68] used the 2D material Ti_2_C_2_T_2_ to study the influence of the confinement effect on the capacitance performance and proved that the dipole polarization of the interlaminar bound water and the negative dielectric constant of the water molecule increase the specific capacitance of the material.

In addition, the lateral dimension also affects the specific capacitance of Ti_3_C_2_T_*x*_. Maleski et al. found when it is 1 μm, the mass ratio capacitance can reach 290 F g^−1^ (2 mV s^−1^) and the volume specific capacitance can reach 1100 F cm^−3^ [82]. When the flake size is 1.47 μm, its capacitance drops to 260 F g^−1^ [82]. This is because that smaller nanosheets have more ion diffusion paths and better ionic conductivity, and larger nanosheets have smaller interface contact resistance and better electron conductivity.

By adjusting the time and power of ultrasonic exfoliating, the size of Ti_3_C_2_T_*x*_ nanosheets can be controlled. The longer the ultrasonic time and the higher the power, the smaller the size of Ti_3_C_2_T_*x*_ nanosheets. It is beneficial to increase ion diffusion sites and tighten the structure. However, the resulting material defects and increased interfacial impedance caused by ultrasonic treatment reduce the conductivity of Ti_3_C_2_T_*x*_ [76, 83]. Anyway, while the smaller size of nanosheets introduces more ion diffusion sites, it also leads to larger interfacial contact resistance and more defects, which are detrimental to the conductivity of Ti_3_C_2_T_*x*_. Therefore, the small size effects or the influence of ultrasonic treatment should be viewed rationally to find the balance.

#### Other Influence

As for the effect of the surface functional group, in addition to the above-mentioned participation in the pseudocapacitance reaction, the decrease in –F is good for increasing the specific capacitance, because the chemical instability of the –F functional group hinders the transfer of electrolyte ions and reduces the specific gravity of Ti ion. Moreover, the introduction of water molecules between layers results in a larger interlayer spacing, which facilitates the contact of the aqueous cations with the electrochemical active sites on the surface. Hu et al. used density functional theory (DFT) calculations to demonstrate that if the surface of Ti_3_C_2_T_*x*_ has only –O functional group, its theoretical specific capacitance can be as high as 1190 F g^−1^ [84]. However, the presence of –F reduces the content of –O, which is the electroactive surface accessible by hydronium ions, thus hindering the transfer of electrolyte ions. If the content of –O drops to 14% of ideal value, its specific capacitance drops to 167 F g^−1^. The increase in –O content is beneficial to the capacitance of Ti_3_C_2_T_*x*_ tending to theoretical value. There are already methods to decline –F for Ti_2_CT_*x*_, such as annealing [85], and we believe the same strategies can be applied to Ti_3_C_2_T_*x*_.

As shown above, many factors affect the capacitance property of Ti_3_C_2_T_*x*_ nanosheets. There are some wrap-up problems in the usage of Ti_3_C_2_T_*x*_ electrode materials. The first one is that the residual impure phase of the precursor phase may lower the material quality of the prepared Ti_3_C_2_T_*x*_, resulting in a decrease in its capacitance performance; the second one is that the precursor phase contains TiC because of excessive carbon or too little titanium due to the improper powder proportion in the sintering process; the third one is the formation of endogenous growth of alpha-alumina in the sintering process of Ti_3_AlC_2_ due to higher oxygen concentration during preparation. Since hydrofluoric acid etching does not affect these two impurities, the specific surface area of Ti_3_C_2_T_*x*_ will be reduced. Since aluminum has a strong adsorption capacity for oxygen, oxygen must be strictly isolated during the preparation of Ti_3_AlC_2_ to avoid these two impurities [86]. The use of a conductive agent such as acetylene black during the test can suppress the orientation of the two-dimensional Ti_3_C_2_, provide a channel for the transfer of ions, and reduce the electrical resistance of the material as a conductive agent [86].

## Enhancing Capacitance

From the above description, we can see that the mixed charge storage mechanism of Ti_3_C_2_T_*x*_ determines that it is a candidate for excellent electrode materials. However, there are still some obstacles, such as stacking, oxidation, and re-crushing [87]. Its layered structure prepared by the etching method is loose, and the surface is rich in surface terminations. These negatively charged surface functional groups form a stable force—van der Waals force, which leads to stacking problems. When Ti_3_C_2_T_*x*_ is in contact with water and air, the most oxidized part is the edge of the nanosheet. The –F functional group is gradually replaced by –O. The oxidation products (TiO_2_) could significantly reduce the conductivity of Ti_3_C_2_T_*x*_ and reduce electrolyte ion accessible sites. The increase in temperature also intensifies the tendency of Ti atoms to be oxidized. Since the etching reaction makes the surface of the material loose, the intercalation and deintercalation of the cation during charging and discharging may cause the deformation of the material or even collapse of the structure. At last, it affects the structural stability of the material, showing low rate capability. At present, many efforts have been made to solve these problems. In the following section, we discuss the measure of improving the capacitor property of Ti_3_C_2_T_*x*_, including structure modulation method and composite reinforcement method.

### Structure Modulation Method

Surface modification and film formation of Ti_3_C_2_T_*x*_ are methods commonly used to boost the specific capacitance of Ti_3_C_2_T_*x*_. Currently, Ti_3_C_2_T_*x*_ aerogel also gains wide attention. In the following, these three types of strategies to increase specific capacitance of Ti_3_C_2_T_*x*_ are discussed in detail.

#### Surface Modification

The –F functional group on the surface of Ti_3_C_2_T_*x*_ inhibits it to obtain the desired specific capacitance. Since the Ti–F bond has lower strength at higher pH, KOH is used to alkalize Ti_3_C_2_T_*x*_, using –OH to replace –F. According to the study of the thermal stability of Ti_3_C_2_T_*x*_, it can be seen the surface hydroxyl group can be removed by annealing treatment, and finally, the specific gravity of the –O functional group of Ti_3_C_2_T_*x*_ is obtained, which owns more electrochemical active sites. In addition, the annealing treatment also enhances the structural order of the material, which is advantageous for increasing the conductivity. Li et al. optimized Ti_3_C_2_T_*x*_ through KOH alkalization and heat treatment to obtain better gravimetric capacitance of 517 F g^−1^ (1 A g^−1^) which is about 211% of original Ti_3_C_2_T_*x*_ [88]. Coincidentally, Zhang et al. modified Ti_3_C_2_T_*x*_ film using the same strategy achieving high specific capacitance of 496 F g^−1^ (2 mV s^−1^) in the H_2_SO_4_ electrolyte [89].

#### Film Formation

**Fig. 5:**
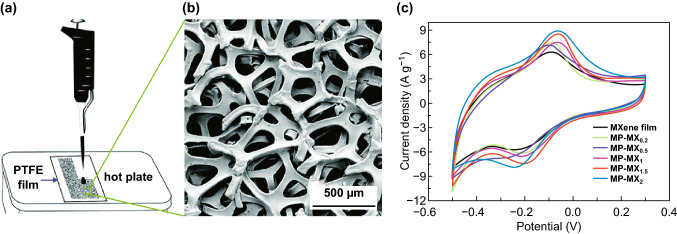
Ti_3_C_2_T_*x*_ film. **a** Schematic of the preparation of the nanoporous Ti_3_C_2_T_*x*_ films. **b** SEM image of nickel foam [92]. **c** CV curves of Ti_3_C_2_T_*x*_ film and modified nanoporous films at a scan rate of 10 mV s^−1^. MP-MX_x_ means the nanoporous Ti_3_C_2_T_*x*_ film is obtained, where x is the mass ratio of Fe(OH)_3_ hybrid [94]. Reproduced with permission from Refs. [92, 94]

Membrane electrodes, as a common electrode, have been studied extensively in Ti_3_C_2_T_*x*_ due to their better structural stability than powder materials. Spin coating, vacuum filtration, rolling, etc., are used to prepare thin film electrodes [90]. Some researchers deposit the fewer layers of Ti_3_C_2_T_*x*_ organic colloid on the foamed nickel by electrophoretic deposition [91]. This method reduces the aggregation of Ti_3_C_2_T_*x*_, increases the accessibility of the electrolyte ions, and improves the conductivity of the material. Ultimately, the specific capacitance of 140 F g^−1^ was obtained in the KOH electrolyte. Electrophoretic deposition is beneficial to the uniform penetration of Ti_3_C_2_T_*x*_ into the three-dimensional porous structure of foamed nickel. However, if an aqueous solution is used, it will cause oxidation of Ti_3_C_2_T_*x*_, which can be effectively avoided by using organic solvent. Hu et al. dripped the suspension on the foamed nickel and heated it at 50 °C to form a Ti_3_C_2_T_*x*_ film, covering the foamed nickel skeleton, as shown in Fig. 5a, b [92]. This film synthesis method is called the dropping-mild baking method (DMB). The DMB method can be applied for the preparation of film-like materials with complex substrate shapes and controllable thickness. By controlling the amount of active materials in the suspension, the thickness of the film can be adjusted. When the mass loading of the film is lower, the capacitance of Ti_3_C_2_T_*x*_ film is better, which means this method is not suitable for constructing thick electrode. The better capacitance contributes to the low impedance of ion percolation and structural stability in an acidic solution. At the same time, the structural stability can be proved by the capacitance of the film which did not have significant attenuation after 10,000 cycles [92]. The quasi-core–shell structure is formed on the foamed nickel skeleton by the negatively charged Ti_3_C_2_T_*x*_ and the positively charged polyethyleneimine layer-by-layer electrostatic self-assembly technique, which has a larger effective contact area and fast conductive path compared with the conventional deposition method [93].

The foamed nickel skeleton forms a conductive channel to prevent re-crushing of Ti_3_C_2_, and wrinkles appear on the surface of the covered Ti_3_C_2_. Large number of mesopores provide more active sites while inhibiting the self-stacking of Ti_3_C_2_. However, the presence of metal collector catalyzes the decomposition of water, which decreases the voltage window of the Ti_3_C_2_T_*x*_ material and causes an increase in electrode weight and manufacturing cost. All these limit the mass loading of active material. The appearance of porous foam-like membrane electrode can effectively solve this problem. After mixing with hydrazine hydrate, it is found that Ti_3_C_2_T_*x*_ formed connected porous materials under high temperature and high pressure. And the reduced atmosphere formed by hydrazine hydrate significantly prevents Ti_3_C_2_T_*x*_ oxidation and inhibits the Ti_3_C_2_T_*x*_ stacking. Shortening the ion diffusion path is beneficial to the intercalation–deintercalation of electrolyte ions. Shi et al. [44] called the connected structure Ti_3_C_2_T_*x*_-foam electrode which has specific capacitance of 122.7 F g^−1^ (5 mV s^−1^) in 1 M KOH electrolyte. However, during the heat treatment, the intercalation water will have a deintercalation layer to reduce the interlayer spacing, and the decrease in the hydrophilic group content will affect the hydrophilicity of Ti_3_C_2_T_*x*_ and reduce the ion permeation of the aqueous electrolyte. To maintain the density of the membrane electrode, nanopores are formed on the surface of the Ti_3_C_2_T_*x*_ membrane by inserting and removing Fe(OH)_3_ nanoparticles, thereby increasing the transport efficiency of ions [94]. Since Fe(OH)_3_ nanoparticles are positively charged, they can be easily bonded to Ti_3_C_2_T_*x*_, and the –F and –OH functional groups are removed by heat treatment to increase the specific gravity of Ti and the density of the film. The porous nanofilm formed by this method exhibits a wrinkle shape, suppresses the stacking of Ti_3_C_2_T_*x*_, improves the connectivity between the nanopores, increases the interlayer spacing, and facilitates the diffusion of ions to maintain electrochemical performance under high load conditions. Figure 5c shows the improvement between Ti_3_C_2_T_*x*_ films and modified films [94]. Indeed, even if the mass loading reaches practical level (11.2 g cm^−2^), it still retains an applicable capacitance (749 F cm^−3^).

#### Ti_3_C_2_T_*x*_ Aerogel

**Fig. 6:**
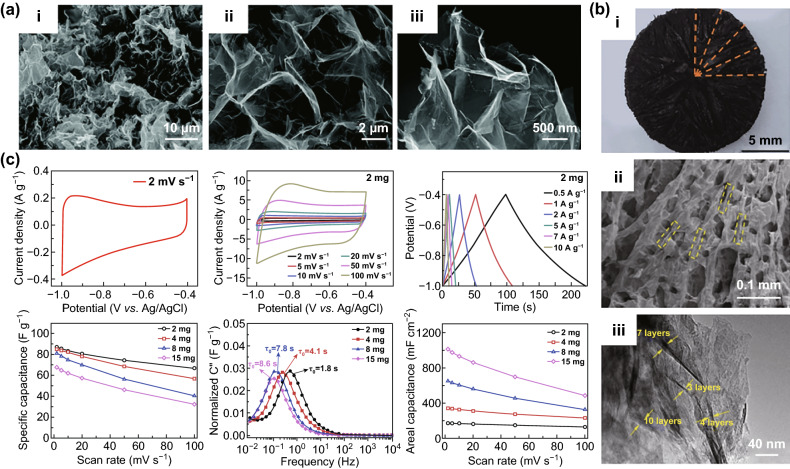
Ti_3_C_2_T_*x*_ aerogel. **a** SEM image of the Ti_3_C_2_T_*x*_ aerogel with different magnifications [40]. **b** Cross-sectional view of Ti_3_C_2_ aerogel, SEM image of Ti_3_C_2_ aerogel, and TEM of void walls. **c** CV and GCD curves of Ti_3_C_2_ aerogel, specific capacitance with different mass loadings, progression of the imaginary (C″) parts of the stack capacitance of Ti_3_C_2_ aerogel and areal capacitance with different mass loadings [96]. Reproduced with permission from Refs. [40, 96]

The preparation of Ti_3_C_2_T_*x*_ into aerogel material can effectively increase the redox active sites to solve the problem of stacking, which can help its specific capacitance get closer to the theoretical value [67, 95]. Wan et al. utilized the overflow of ammonia gas to intercalate N atoms [40]. After that, the nanolayer wrinkles formed nanopores to suppress the stacking of nanolayers (Fig. 6a). The strong bondings on the surface such as N–Ti and O–Ti ensure a larger size for the nanolayer to enhance the stability of the structure, while increasing the mass loading of the electrode and decreasing the specific capacitance value. The use of freeze wet gel instead of solvent produces large number of mesopores and connected structures to increase the specific surface area [96]. The extrusion effect of ethylenediamine (EDA) is used to assist the formation of Ti_3_C_2_ aerogel to form –NH and –NH_2_ functional groups, and the cross section is as shown in Fig. 6b [96]. The substitution of the oxygen-containing functional group facilitates the formation of the nitrogen-containing functional group doping and the gel porous structure, but the EDA belongs to the fatty material, which muffles the redox reaction and reduces the cycle stability (Fig. 6c). After Ti_3_C_2_T_*x*_ formed an aerogel film by vacuum-assisted filtration, the Ti_3_C_2_T_*x*_ ion gel was prepared by immersion in an ionic liquid and vacuum drying, and large number of wrinkles appeared on the surface to increase the contact sites of the cation [97]. Due to the wide variety of ionic liquids, gel films prepared by this method have great application prospects.

### Composite Method

#### Conductive Polymers (CPs)

**Fig. 7:**
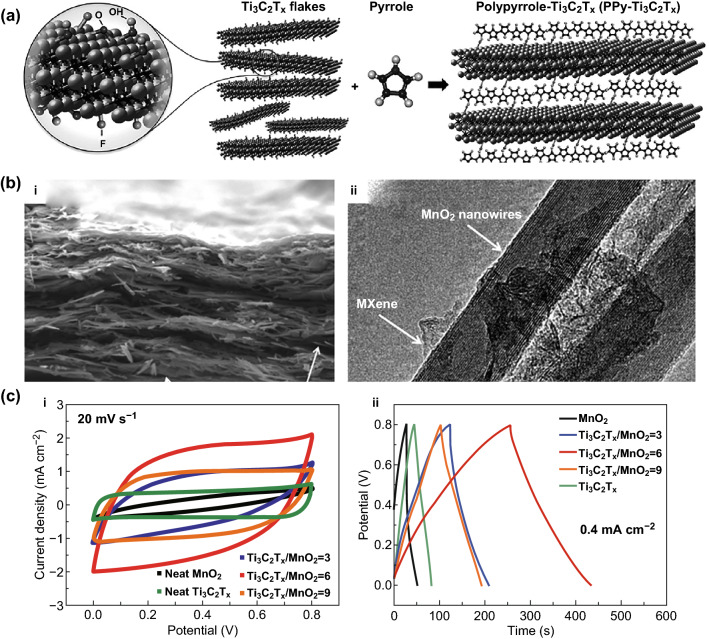
Ti_3_C_2_T_*x*_ composited with PPy and MnO_2_. **a** Schematic illustration of pyrrole polymerization using Ti_3_C_2_T_*x*_. The surface groups on the latter contribute to the polymerization process [99]. **b** Cross-sectional SEM image, and TEM image of Ti_3_C_2_T_*x*_/MnO_2_ nanowires. **c** CV and GCD curves for different samples about Ti_3_C_2_T_*x*_/MnO_2_ nanowires [104]. Reproduced with permission from Refs. [99, 104]

To date, large number of researches have used electrostatic self-assembly or hydrogen bonding to combine CPs with Ti_3_C_2_T_*x*_ to form a layered alternating sandwich structure [16, 98, 99]. The single-layer hydrated intercalation changes the content of functional groups on the surface of Ti_3_C_2_T_*x*_, reducing –F, –OH, and interlayer water [100]. Since the pillaring effect increases the interlayer spacing of Ti_3_C_2_T_*x*_, the bulk density of hydrazine hydrate is low and therefore does not hinder the active sites contacted with electrolyte ions. However, the intercalation of hydrazine hydrate is reversible and easily destroyed, so the structural stability is reduced after the introduction of hydrazine hydrate. PPy is one of the most common conductive polymers. The functional groups on the surface of NH and Ti_3_C_2_T_*x*_ on the pyrrole ring form an alternating layered structure through hydrogen bonding, which is beneficial to the directional growth of PPy. PPy intercalation increases the interlayer space of Ti_3_C_2_T_*x*_ (Fig. 7a), while this ordered structure maintains the high conductivity of Ti_3_C_2_T_*x*_, providing a path for electrolyte ion penetration [99]. Gogotsi et al. proposed a method for the polymerization of pyrrole using acidity, avoiding the damage of oxidizing agents. But the introduction of PPy causes a slight decrease in the density of the material, and the presence of undoped PPy causes internal impedance to increase as its content increases. Wu et al. proposed low-temperature chemical oxidative polymerization forming well-arranged and uniformly distributed PPy nanoparticles [101]. It is deposited on the Ti_3_C_2_T_*x*_ nanosheet by hydrogen bonding and electrostatic interaction (N on the PPy and the –F on the Ti_3_C_2_T_*x*_ form electrostatic force), forming a synergistic effect. Ti_3_C_2_T_*x*_ weakens the aggregation of PPy particles and improves the structural stability. At the same time, the intercalation of PPy expands the interlayer spacing of Ti_3_C_2_T_*x*_ and parallels lamellar structure, shortening the diffusion path of electrolyte ions. In addition to forming a sandwich structure, the three-dimensional conductive network also has a good application prospect. One-dimensional PPy nanowires and Ti_3_C_2_T_*x*_ nanoparticles form a three-dimensional porous structure on a foamed nickel substrate, inhibiting the aggregation between Ti_3_C_2_T_*x*_, exposing more amorphous carbon, enhancing charge transfer rate, and introducing PPy monomer intercalation or polymerization to form an open structure around MXene, which spreads the interlayer spacing and crystallinity of Ti_3_C_2_T_*x*_ [102].

Most methods of increasing specific capacitance are based on the premise of sacrificing density. The drawback of this strategy is to reduce the volumetric capacitance and energy density of the material. The oxidation of aniline with –O and –OH on the surface of Ti_3_C_2_T_*x*_, the hydrophilicity of the product, causes the composite to self-polymerize, and the polyaniline (PANI) is electrochemically deposited onto the surface of Ti_3_C_2_T_*x*_ of the composite material which causes wrinkle on the surface [43, 103]. PANI increases the layer spacing of Ti_3_C_2_T_*x*_ to accelerate the entry of ions into the electrode material, increases the contact point of the electrolyte ions with the redox active site, and improves the conductivity of the material. In addition, the –OH and –F are reduced, and N atoms appear on the surface of the Ti_3_C_2_T_*x*_/PANI composite film. Due to the high conductivity and ionic conductivity of the film, good specific capacitance of 371 F g^−1^ at scan rates of 2 mV s^−1^ and great capacitance retention after 10,000 cycles can be obtained even with thick electrodes (up to 45 microns) [103]. This has further broadened the use of Ti_3_C_2_T_*x*_ in portable or self-powered devices.

#### Transition Metal Oxides (TMOs)

TMOs have the characteristics of low cost, high theoretical specific capacitance, environmental friendliness, and stable electronic transportation. The combination of Ti_3_C_2_T_*x*_ and TMOs can effectively improve its poor conductivity. Zhou et al. [104] mixed the one-dimensional MnO_2_ and Ti_3_C_2_T_*x*_ into an ink-like material, forming a wrinkled silk-like film by solution processing, and the surface of both MnO_2_ and Ti_3_C_2_T_*x*_ was negatively charged and electrostatically repelled to form a uniform colloid (Fig. 7b, c). The composite structure produces a significant synergistic effect. The insertion of MnO_2_ nanowires into the Ti_3_C_2_T_*x*_ film increases the specific surface area. At the same time, the hybrid structure of the one-dimensional material and the two-dimensional material significantly increases the interlayer spacing and facilitates ion transport. The MnO_2_ nanowires are interconnected by Ti_3_C_2_T_*x*_ to increase the conduction speed of electrons.

The positively charged MnO_2_ nanosheet and the negatively charged Ti_3_C_2_T_*x*_ form a composite film by electrostatic self-assembly, and the electrostatic attraction makes a close relationship between the two materials and enhances the interface electron conduction capability [98]. The self-assembled structure inhibits the phenomenon of Ti_3_C_2_T_*x*_ stacking, which is beneficial to the diffusion of ions. MnO_2_ immobilized on the nanosheet promotes the uniform distribution of the ions on the surface of the electrode, improves the structural stability of the electrode, and prevents re-crushing. MnO_2_ is intercalated into Ti_3_C_2_T_*x*_ by liquid deposition and heat treatment. Ti_3_C_2_T_*x*_ maintains its layered structure, and MnO_2_ nanoparticles inhibit the stacking of the sheets [106]. The layer spacing of the material is larger than the diameter of the electrolyte cations, thereby increasing the ion intercalation. The area of the layer increases the channel for diffusion of electrolyte ions. MnO_2_ forms a layered porous structure on the surface of Ti_3_C_2_, and the uniform distribution of MnO_2_ on the surface increases the effective surface area.

In addition to MnO_2_, there are many other transition metal oxide materials combined with Ti_3_C_2_T_*x*_ to enhance its capacitance performance, like NiO [107], MoO_3_ [108], WO_3_ [39], Fe_2_O_3_ [109], TiO_2_, and so on [110, 111]. Furthermore, Wang et al. [112] reported a Ti_3_C_2_T_*x*_ composited with TMO containing two transition metal elements. According to this report, nickel and molybdenum are attached to the surface of Ti_3_C_2_T_*x*_ to form a flower-like connected nanostructure by using the hydrothermal method and intercalation [112]. This porous structure basically has no stacking problem commonly found in 2D materials and significantly increases the specific surface area of the material and accelerates the electrolyte. The specific area of flower-like structure is from 24.15 m^2^ g^−1^ (Ti_3_C_2_T_*x*_) to 152.3 m^2^ g^−1^ [112]. During the conduction of ions, the thickness of the sheet is reduced and the pitch becomes larger to expose more electrochemically active sites. Hydrophilic Ti_3_C_2_T_*x*_ facilitates the wettability of the electrode and shortens the path of electrolyte ion diffusion. The Ti_3_C_2_T_*x*_/NiMoO_4_ heterojunction prepared with this method has a specific capacitance of up to 1364 F g^−1^ in the 3 M KOH electrolyte [112]. The research ideas of these methods are to increase the specific capacitance by the synergistic effect and TMO pseudocapacitance performance.

#### Carbon-Based Materials

**Fig. 8:**
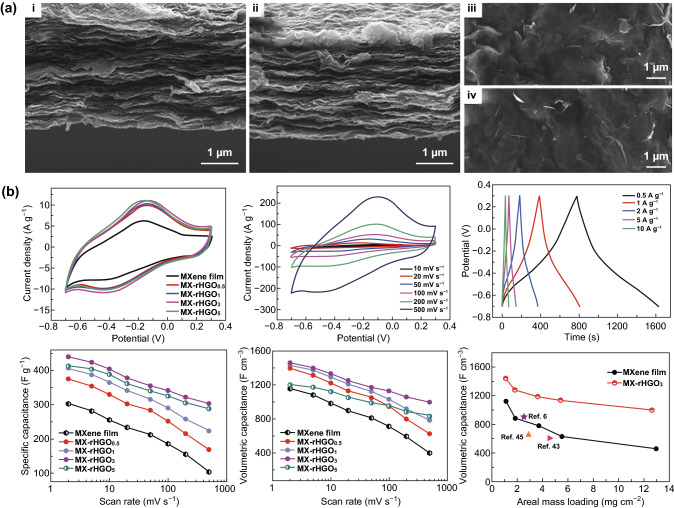
Ti_3_C_2_T_*x*_-rHGO nanoporous network. a Cross section (i, ii) and SEM images (iii, iv) of Ti_3_C_2_T_*x*_ film (i, iii) and Ti_3_C_2_T_*x*_-rHGO films (ii, iv). **b** CV and GCD curves of Ti_3_C_2_T_*x*_ films and Ti_3_C_2_T_*x*_-rHGO and effects of areal mass loading on the volumetric capacitance. MX-rHGO_*x*_, where x is the percentage of the weight of holey graphene oxide in the mixture. Reproduced with permission from Ref. [117]

There are two ways to improve the performance of MXene capacitors, i.e., forming a column effect or introducing space to increase the layer spacing [29, 113–115]. The exfoliated Ti_3_C_2_T_*x*_ and graphene oxide (GO) mixture was vacuum-filtered into a film. After reduction heat treatment, rGO/Ti_3_C_2_T_*x*_ hybrid film was prepared. The larger layers of rGO link the dispersed Ti_3_C_2_T_*x*_ layers and remove some surface functional groups [116]. As a result, the conductivity of Ti_3_C_2_T_*x*_ increases. Meanwhile, Ti_3_C_2_T_*x*_ reduces the aggregation of rGO to improve ion transport efficiency of hybrid films. Moreover, the additional polymer binder increases the accessibility of ions and reduces the internal resistance of the composite membrane. During the vacuum heating and reduction process, the oxygen surface of the GO surface is removed to cause wrinkles on the surface, which increases the specific surface area. Reduced holey graphene oxide (rHGO) forms a high-connectivity nanoporous network with Ti_3_C_2_T_*x*_, as shown in Fig. 8a [117]. Unlike other methods of forming porous composite membranes, this method retains the dense layer to maintain mechanical properties such as flexibility, and the surface area of the material is significantly increased. High volume specific capacitance of 1445 F cm^−3^ and high mass specific capacitance of 438 F g^−1^ were obtained in the 3 M H_2_SO_4_ electrolyte [117]. rHGO provides a large number of active sites, and this structure controls the stacking of Ti_3_C_2_T_*x*_ to enlarge the interlayer spacing. The introduction of graphene has a slight effect on the density of Ti_3_C_2_T_*x*_, but the volumetric capacitance remains at a very high level, and the effect of mass loading on volumetric capacitance is not conducive to the fabrication of thick film electrodes. The CV curves of different mass ratios between Ti_3_C_2_T_*x*_ and rHGO are shown in Fig. 8b.

**Fig. 9:**
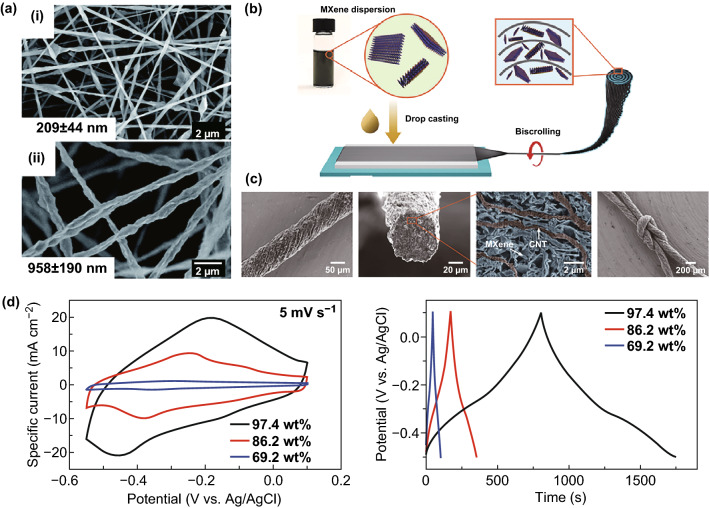
One-dimensional electrode materials of supercapacitors. **a** Morphology of electrospinning Ti_3_C_2_T_*x*_ composite [118]. **b** Schematic of the fabrication process of Ti_3_C_2_T_*x*_ fiber using biscrolling method. **c** Surface and cross-sectional morphologies of biscrolling Ti_3_C_2_T_*x*_ fiber. **d** CV curves obtained at 5 mV s^−1^ and GCD curves obtained at 2 mA cm^−2^ [120]. Reproduced with permission from Refs. [118, 120]

Fiber electrodes have become a research hot spot in the field of Ti_3_C_2_T_*x*_, especially Ti_3_C_2_T_*x*_ and carbon-based materials. Ti_3_C_2_T_*x*_/carbon fiber is prepared by electrospinning Ti_3_C_2_T_*x*_ and recarburizing PAN fiber structure. Compared with the wrapped one-dimensional electrode material, the connection between two materials is stronger, so there is no hazard of falling off of active material, helping to maintain a good conductivity and electrode stability. However, electrospinning (Fig. 9a) has the disadvantages of uneven distribution of Ti_3_C_2_T_*x*_ and small mass loading of active material [118]. The composite of MXene and carbon tube is scrolled into a fiber-shaped spiral structure [119]. With mechanical incompatibility between carbon tube film and Ti_3_C_2_T_*x*_ nanosheet, Ti_3_C_2_T_*x*_ is closely related to CNTs and has high mechanical robustness and electron conductivity. Therefore, there is a gap between them to facilitate the rapid transport of ions, and the specific surface area is large. The loading of the active material in this method can not only reach a high level, but also cause an increase in the internal resistance of the composite fiber material. Biscrolling approach was used to prepare flexible fibrous materials, that is, Ti_3_C_2_T_*x*_ drop-cast on the surface of carbon tubes and biscrolling into fibers, as shown in Fig. 9b. Ti_3_C_2_T_*x*_ loading can be controlled simply by the adjusting the concentration of the suspension. The uniform distribution of Ti_3_C_2_T_*x*_ (Fig. 9c) forms a lot of vacancies on the surface of the carbon tube to maintain the high conductivity of Ti_3_C_2_T_*x*_ [120]. The electrolyte ions are infiltrated due to strong hydrophilicity. With the increase in mass loading, the specific capacitance of the material increases. When the Ti_3_C_2_T_*x*_ mass loading is up to 98%, the specific capacitances can be as high as 1083 F cm^−3^ (428 F g^−1^) in 3 M H_2_SO_4_ electrolyte, as shown in Fig. 9d.

#### Heteroatomic Doping

**Fig. 10:**
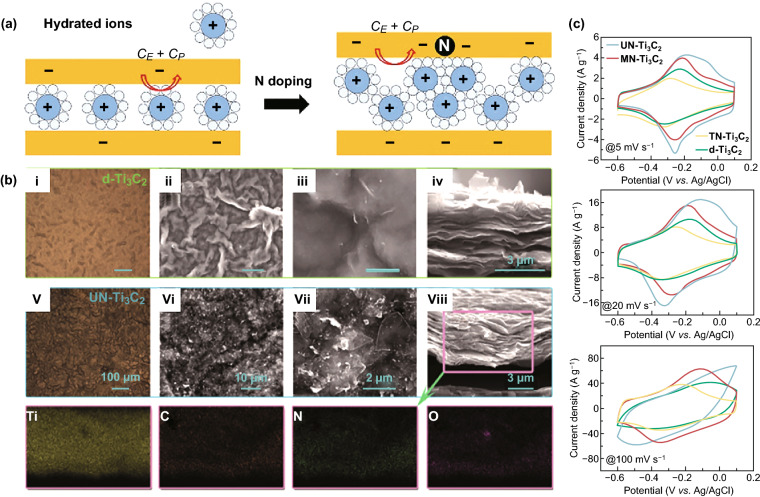
N-doped Ti_3_C_2_T_*x*_. **a** Schematic illustration of charge storage of hydrated electrolyte ions in N-doped Ti_3_C_2_T_*x*_ [122]. **b** Plan view of optical and SEM images of Ti_3_C_2_ and N-doped Ti_3_C_2_T_*x*_ using urea as the nitrogen source (UN-Ti_3_C_2_). **c** CV curves of the Ti_3_C_2_ films and N-doped Ti_3_C_2_ using different method, such as UN-Ti_3_C_2_, MN-Ti_3_C_2_ (nitrogen source is monoethanolamine), TN-Ti_3_C_2_ (solid solution method) [123]. Reproduced with permission from Refs. [122, 123]

Doping on Ti_3_C_2_T_*x*_ surface is a common surface modification method [44]. There are two methods for nitrogen atom doping [121]. One method is to directly anneal in ammonia gas. The N atom replaces the position of C in Ti_3_C_2_T_*x*_, while the heat treatment removes –F functional group. Figure 10a shows the charge storage of hydrated electrolyte ions in N-doped Ti_3_C_2_T_*x*_ [122]. The other method is solvothermal method doping N on Ti_3_C_2_T_*x*_ surface. Compared with in-situ solid solution doping, solvothermal method can increase the specific capacitance of the material by adjusting the type and content of nitrogen source (Fig. 10b) [123]. The fluidity of ethanol and low boiling point assist the nitrogen source to diffuse between the layers. The N-doped Ti_3_C_2_T_*x*_ surface wrinkles, containing rich N active sites and structural defects to form a connected pore structure which is beneficial to the diffusion of hydrogen ions. The surface of the doped material has mesopores and narrow slits, increasing the specific surface area. Since both the nitrogen source and Ti_3_C_2_T_*x*_ contain oxygen functional groups, the oxidation of Ti_3_C_2_ is inevitable in the doping process, which affects the structural stability of the material. Nitrogen doping forms three bonding forms: Quaternary N (N-Q) contributed to N atoms replaces the position of C in the Ti_3_C_2_ lattice to enhance conductivity, pyrrolic N (N-5) undergoes rapid redox reaction (Fig. 10c), and N-Ti bond improves the wettability and structural stability of the electrode material [124–126]. In addition to nitrogen atom doping, N-doped carbon is also a common method [127]. The comparison of those Ti_3_C_2_T_*x*_-based materials is shown in Table 1.

**Table 1:** Comparison of capacitance performance of Ti_3_C_2_T_*x*_-based materials, where *C*_s_ is specific capacitance

Materials	Feature	Electrolyte	*C*_s_	Rate	References
Ti_3_C_2_T_*x*_ aerogels	N_2_ spill forming wrinkles	3 M H_2_SO_4_	438 F g^−1^	10 mV s^−1^	[40]
Freeze-dried Ti_3_C_2_T_*x*_ aerogel	Nanoporous structure and –O replaced by NH, NH_2_	1 M KOH	1012.5 mF cm^−2^	2 mV s^−1^	[96]
Ti_3_C_2_T_*x*_ ion gel	Large number of wrinkles	Ion liquid	70 F g^−1^	20 mV s^−1^	[97]
Ti_3_C_2_T_*x*_/PPy	Polymerization of pyrrole using acidity and no Oxidizing agents	1 M H_2_SO_4_	416 F g^−1^	5 mV s^−1^	[99]
Ti_3_C_2_T_*x*_/PPy nanoparticles	Well-arranged, uniformly distributed PPy nanoparticles	1 M Na_2_SO4	184.36 F g^−1^	2 mV s^−1^	[101]
Ti_3_C_2_T_*x*_/PPy nanowires	Three-dimensional porous structure on a foamed nickel	3 M KOH	610 F g^−1^	0.5 A g^−1^	[102]
Ti_3_C_2_T_*x*_/PANI	–OH and –F are reduced and N atoms appear	3 M H_2_SO_4_	503 F g^−1^ (1682 F cm^−3^)	2 mV s^−1^	[103]
Ti_3_C_2_T_*x*_/MnO_2_ nanowires	Wrinkled silk-like film	PVA/LiCl	205 mF cm^−2^ (1025 F cm^−3^)	1 A cm^−3^	[104]
Ti_3_C_2_T_*x*_/MnO_2_ nanosheet	Composite film forming by electrostatic self-assembly	1 M Na_2_SO_4_	340 F g^−1^	1 A g^−1^	[105]
Ti_3_C_2_T_*x*_/MnO_2_ nanoparticles	Layered porous structure, uniform distribution of MnO_2_	6 M KOH	377 mF cm^−2^	5 mV s^−1^	[106]
rGO/Ti_3_C_2_T_*x*_ hybrid film	Larger size rGO links the dispersed Ti_3_C_2_T_*x*_ layer	6 M KOH	405 F g^−1^ (370 F cm^−3^)	1 A g^−1^	[117]
Ti_3_C_2_T_*x*_/CNTs	Electrospinning method	1 M H_2_SO_4_	205 mF cm^−2^	50 mV s^−1^	[118]
Ti_3_C_2_T_*x*_/CNTs	Biscrolling approach	3 M H_2_SO_4_	428 F g^−1^ (1083 F cm^−3^)	2 mA cm^−2^	[120]
Ti_3_C_2_T_*x*_/CNTs	Scrolling into a fiber-shaped spiral structure	6 M LiCl	19.1 F cm^−3^	1 A cm^−3^	[119]
N-doped Ti_3_C_2_T_*x*_	Anneal in ammonia gas directly	1 M H_2_SO_4_	192 F g^−1^	1 mV s^−1^	[122]
N-doped Ti_3_C_2_T_*x*_	Solvothermal method (urea as nitrogen source)	3 M H_2_SO_4_	927 F g^−1^ (2836 F cm^−3^)	5 mV s^−1^	[123]

The synthesis of N-doped carbon-decorated Ti_3_C_2_T_*x*_ complex by dopamine self-polymerization and calcination carbonization reaction effectively inhibits the Ti_3_C_2_T_*x*_ stack from accommodating more electrolyte ions [128]. The nitrogen-doped carbon layer has a high specific surface area. Ti_3_C_2_T_*x*_ provides higher conductivity and inhibits the agglomeration of nitrogen-doped carbon. However, due to the higher heat treatment temperature, a large amount of TiO_2_ nanoparticles are attached to the surface of the material. It is benefical to form more Ti-N bonds by increasing the N doping amount to improve the stability of the structure and increase the active suface. However, if the content is too high, the internal carbon layer cannot contact the electrolyte ions and cannot participate in the electrochemical process. The charge and discharge process will reduce the specific capacitance.

#### Other Materials

The hybridization of Ti_3_C_2_T_*x*_ with other materials into a two-dimensional heterostructure is beneficial to increase the specific surface area of the material and expose more active sites. The chemical bath deposition method was used to deposit a petal-like bismuth oxychloride (BiOCl) on the surface and between the Ti_3_C_2_T_*x*_ nanosheets to increase the interlayer spacing of the material, which significantly increases the surface area of Ti_3_C_2_T_*x*_ [129]. The high conductivity of BiOCl leads to rapid electron transfer and contribution of faradic reaction, and the surface of the composite membrane produces a lot of mesopores to increase the accessible sites of electrolyte ions [129]. In addition, the TiO_2_ particles formed by the oxidation reaction were also decomposed during the electrochemical deposition process. Ni–Co–Al layered double hydroxide (LDH) has high theoretical specific capacitance due to the two valence forms of Ni^2+^ and Co^2+^. Zhao et al. [13] synthesized the Ti_3_C_2_T_*x*_/Ni–Co–Al LDH heterostructures by electrostatic self-assembly, and the face-to-face contact facilitates electronic transportation.

## Conclusions and Perspectives

MXene, as an emerging material compared with carbon materials, has aroused numerous attentions, especially in energy storage field. Among them, Ti_3_C_2_T_*x*_ has received the most attention. In this review, we compare different etching processes and the methods for accelerating exfoliation of Ti_3_C_2_T_*x*_. We sincerely hope that this can guide researchers to propose safer and simpler preparation process. Meanwhile, the capacitance behaviors by the two energy storage mechanisms in Ti_3_C_2_T_*x*_ are summarized, which are cation intercalation leading to redox reaction and ion electrosorption, respectively. Influencing factors and obstacles are analyzed via electrochemical mechanism. Furthermore, we summarize the recent strategies of enhancing capacitance performance, such as film formation, synergistic effect, and heteroatom doping which lay the ground for further research to increase specific capacitance.

Based on this review, we point out the following conjectures on Ti_3_C_2_T_*x*_ future research direction:

1. Although there are many strategies used in preparing Ti_3_C_2_T_*x*_, the problem of low yield of Ti_3_C_2_T_*x*_ remains unsolved and the reason is still unclear. In addition, the traditional etching processes in preparation own many security risks. Those obstacles hinder mass production and commercialization of Ti_3_C_2_T_*x*_.
2. Thinner electrode limits the value of the material, but most improvement strategies are unsuitable for the construction of thick film. So, the electrochemical performance of thick film electrodes requires further investigation.
3. Heteroatom doping, especially N-doping, has been applied as a significant measure to improve the specific capacitance of Ti_3_C_2_T_*x*_, and many studies have proved that wider layer spacing can be obtained after N-doping. However, the mechanism of increasing interlayer space caused by N-doping requires further exploration.
4. The oxidation of Ti_3_C_2_T_*x*_ is one of the most common problems. However, there are few ways to avoid it besides cryogenic storage. Non-conductive TiO_2_ formed by oxidation on the surface of Ti_3_C_2_T_*x*_ seriously affects capacitance performance, especially at the high voltages. Therefore, it is urgent to propose new feasible strategies to prevent the oxidation of Ti_3_C_2_T_*x*_.
5. Currently, Ti_3_C_2_T_*x*_ is mostly used as a negative electrode material for energy storage components, and its application as a positive electrode material needs to be further broadened.
6. Specific capacitance of Ti_3_C_2_T_*x*_ is still far from ideal value, so there is still much room for improvement in the regulation of surface functional groups.
